# Fear Extinction as a Model for Synaptic Plasticity in Major Depressive Disorder

**DOI:** 10.1371/journal.pone.0115280

**Published:** 2014-12-29

**Authors:** Marion Kuhn, Nora Höger, Bernd Feige, Jens Blechert, Claus Normann, Christoph Nissen

**Affiliations:** 1 Department of Psychiatry and Psychotherapy, University Medical Center Freiburg, Freiburg, Germany; 2 Division of Clinical Psychology, Psychotherapy, and Health Psychology, Department of Psychology, University of Salzburg, Salzburg, Austria; Radboud University, Netherlands

## Abstract

**Background:**

The neuroplasticity hypothesis of major depressive disorder proposes that a dysfunction of synaptic plasticity represents a basic pathomechanism of the disorder. Animal models of depression indicate enhanced plasticity in a ventral emotional network, comprising the amygdala. Here, we investigated fear extinction learning as a non-invasive probe for amygdala-dependent synaptic plasticity in patients with major depressive disorder and healthy controls.

**Methods:**

Differential fear conditioning was measured in 37 inpatients with severe unipolar depression (International Classification of Diseases, 10^th^ revision, criteria) and 40 healthy controls. The eye-blink startle response, a subcortical output signal that is modulated by local synaptic plasticity in the amygdala in fear acquisition and extinction learning, was recorded as the primary outcome parameter.

**Results:**

After robust and similar fear acquisition in both groups, patients with major depressive disorder showed significantly enhanced fear extinction learning in comparison to healthy controls, as indicated by startle responses to conditioned stimuli. The strength of extinction learning was positively correlated with the total illness duration.

**Conclusions:**

The finding of enhanced fear extinction learning in major depressive disorder is consistent with the concept that the disorder is characterized by enhanced synaptic plasticity in the amygdala and the ventral emotional network. Clinically, the observation emphasizes the potential of successful extinction learning, the basis of exposure therapy, in anxiety-related disorders despite the frequent comorbidity of major depressive disorder.

## Introduction

Major depressive disorder (MDD) is a substantive personal, societal and economic problem. According to the World Health Organization, MDD is the leading cause of years of life lived with disability worldwide (YLD index [1]). Nevertheless, the understanding of MDD pathophysiology is fragmentary [2], and less than half of the patients with MDD achieve sustained remission with optimized treatment [3]. The predominance of the monoamine hypothesis has – despite some progress over the past decades – more recently been associated with an innovation crisis in the neurobiology of MDD [4]. The *neuroplasticity hypothesis* of depression might have the potential to resolve this crisis and to lead to the development of innovative and more effective treatments [5].

The neuroplasticity hypothesis of MDD proposes a dysfunction of neural plasticity – the basis for learning, memory and adaptive behavior [6] – as a principle pathomechanism for the clinical symptoms of the disorder [7]–[10]. Specifically, synaptic plasticity, the activity-dependent refinement of the strength of neurotransmission across synapses, might be altered in MDD. Synaptic plasticity is a key functional mechanism and is closely linked to the structural modifications of neural networks, including changes in the number of synapses, spines and dentrites, neurogenesis, brain metabolism and volume, and function on the behavioral level.

Synaptic plasticity in MDD appears to be differentially altered in the two major neural systems of emotion processing: the dorsal executive system and the ventral emotional system [11], [12]. In the dorsal executive system that includes the hippocampus and broad cortical areas, a decrease of neural plasticity has been reported in animal models of depression and patients with MDD. Thus, studies indicate decreased synaptic long-term potentiation (LTP) in the hippocampus of rats in an animal model of depression [13]. In the chronic mild stress animal model of depression, synaptic long-term depression (LTD) in the hippocampus was facilitated, and neurogenesis in the dentate gyrus was reduced [14]. In patients with MDD, reductions in neuronal cell bodies and neuropil in the postmortem hippocampus [15], reduced glia density in the prefrontal cortex [16], reduced hippocampal volumes [17]–[21], reduced cerebral blood flow [22], impaired plasticity in the visual cortex [9], and attenuated hippocampus-dependent memory consolidation [23] have been observed. In contrast, neural plasticity in MDD might be enhanced in the ventral emotional system, composed of the amygdala [12], with enlarged volume [24], [25] and increased glucose metabolism in the amygdala [26] and enhanced amygdala-dependent learning in patients with MDD [23]. Altogether, these findings support the idea of a hypoplastic executive system and a hyperplastic emotional system in MDD.

On the molecular level, these changes in the two systems of emotion processing in MDD might be explained by the contrasting effects of chronic stress as a key mediator of MDD [27]. Chronic stress decreases the expression of the brain-derived neurotrophic factor (BDNF), a key factor for long-term synaptic plasticity, in the hippocampus [28], elicits dendritic atrophy in hippocampal CA3 pyramidal neurons [29], and impairs N-methyl-D-aspartate receptor (NMDAR)-dependent LTP in the hippocampus [13], [30]. On the contrary, chronic stress leads to hypertrophy and hyperactivity in the neurons of the basolateral amygdala [29], [31] and enhances NMDAR-dependent LTP in the amygdala [32]. Whereas hippocampus-dependent plasticity has been relatively well described, amygdala-dependent plasticity remains to be further characterized in MDD.

The most widely accepted model of amygdala-dependent synaptic plasticity is fear conditioning [33]. The association between a conditioned stimulus (CS+) and unconditioned stimulus (US) formed in fear acquisition involves local synaptic LTP in the lateral amygdala [33]–[35]. In a previous paradigm, we found enhanced fear acquisition in patients with MDD [23]. This paradigm involved a complex CS-US contingency to prolong the acquisition process and to detect subtle group differences in acquisition speed and strength. The observation was in line with enhanced activity in a ventral emotional network and increased amygdala-dependent LTP in MDD.

Although investigating how fear is acquired is of great relevance, it is equally or even more important to elucidate how acquired fear can be diminished. One approach for reducing acquired emotional associations is extinction learning. Extinction learning refers to the reduction of fear to a CS+ previously linked to an aversive US when the CS+ is repeatedly presented without the US.

To date, it is widely accepted that extinction learning is a new learning process, forming a novel memory trace that coexists with the previously acquired CS-US association [36]. This view is based on observations on the behavioral level that extinguished fear responses to a CS+ can spontaneously recover with the passage of time (spontaneous recovery; [37]–[39]), return with a context change after extinction (renewal; [40], [41]), or reinstate after receiving unsignaled USs in the same context of learning (reinstatement; [42], [43]). These phenomena provide evidence that extinction does not erase the original CS-US association.

In addition, neurobiological studies indicate that extinction learning involves the formation of a novel memory trace. The extinction of fear, like the initial acquisition, emerges from NMDAR-dependent LTP in the basolateral amygdala [44]. Specifically, the infusion of the NMDAR antagonist AP5 (D, L-2-amino-5-phosphonovaleric acid) into the basolateral amygdala of rats blocks fear extinction [45]. Conversely, injection of the partial NMDA receptor agonist D-cycloserine into the same region facilitates fear extinction [46], [47]. Together, these data identify synaptic LTP in the basolateral amygdala as the central neural mechanism and site of fear extinction learning.

The primary aim of this study was to investigate fear extinction learning as a non-invasive probe for amygdala-dependent synaptic plasticity in humans to further test the neuroplasticity hypothesis of MDD. A simple acquisition paradigm was used to induce robust fear acquisition in patients with MDD and healthy controls. Extinction learning was subsequently induced through an immediate extinction paradigm. We hypothesized enhanced fear extinction learning in patients with MDD compared to healthy controls, indicative of enhanced amygdala-dependent synaptic plasticity in MDD.

## Methods and Materials

### Participants

The final sample consisted of 37 patients meeting ICD-10 criteria for severe unipolar depression and 40 healthy controls, matched for sex, age and years in school (*N*  = 77). A total of 45 patients and 48 healthy controls had initially been screened for potential participation. Five patients and five healthy controls were excluded after the screening session because they did not meet the inclusion criteria. Data from three patients and three healthy controls could not be analyzed due to recording failures. The study was approved by the local Ethics Committee of the University Medical Center Freiburg. According to the guidelines of the Ethics Committee, all participants provided written informed consent prior to participation. Inpatient participants and healthy controls were given the option to opt out or cease their participation at any point during the study.

Patients with MDD were inpatients in the Department of Psychiatry and Psychotherapy, University Medical Center Freiburg, and were receiving psychotherapy and stable medication (>2 weeks) with one or more antidepressants at the time of participation. Routine blood tests and magnetic resonance scanning were used to exclude organic affective disorders. None of the patients received any antipsychotics, benzodiazepines, or other compounds that act on the central nervous system. Other lifetime axis I or II disorders were exclusion criteria. Twenty-six of the 37 patients presented with a recurrent disorder. The mean score of the 21-item Hamilton Rating Scale for Depression (HAMD) was 28.6±5.7. The average number of episodes was 3.1±2.4, and the duration of the current episode was 42.5±71.4 weeks. The average age of MDD onset was 27.4±12.3 years, the average total lifetime duration of illness at the time of participation was 10.6±9.5 years, and the duration of the current antidepressive medication was 18.8±33.1 weeks.

Healthy controls were recruited from the community and compensated for their participation. They had no history of a mental or other relevant disorder (lifetime) and were free of any psychoactive medication. All participants were right-handed and had normal or corrected-to-normal vision. The participants did not consume alcohol or caffeine during the study.

### Study Design

All participants underwent a classical fear acquisition and extinction paradigm. To limit circadian effects, all experiments were conducted between 4∶00 pm and 6∶00 pm. To control for general neuropsychological effects, short-term memory (Digit Span test of the Hamburg-Wechsler Intelligence Scale for Adults) and alertness (Test for Attentional Performance, TAP) were assessed prior to the fear conditioning paradigm.

### Fear Conditioning Procedure

**Figure 1:**
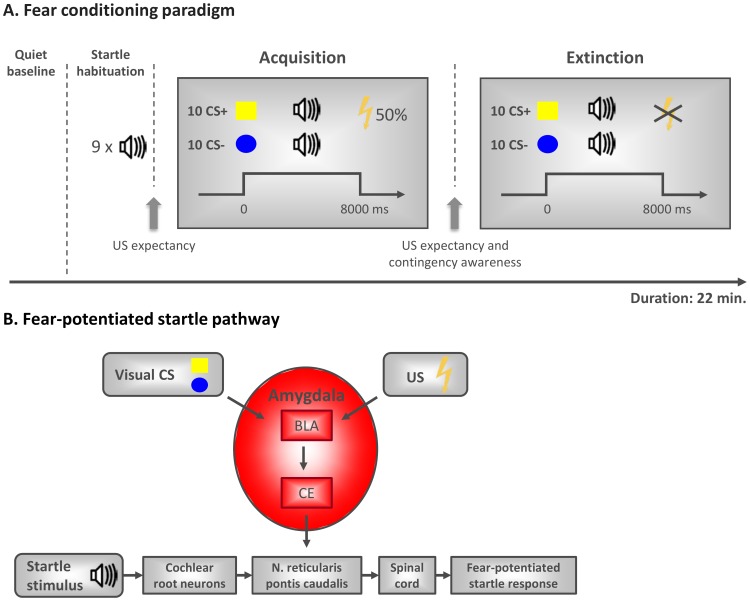
A. Fear-potentiated startle pathway. The primary acoustic startle pathway comprises only a few synapses involving the cochlear root neurons, neurons in the nucleus reticularis pontis caudalis, and motoneurons in the spinal cord that innervate the musculus orbicularis oculi and illicit the startle response. This pathway is critically modulated by the output of the amygdala. The basolateral amygdala (BLA) receives and integrates sensory information from multiple sources; here, the inputs of the visually presented conditioned stimuli (CS+ and CS−; yellow square, blue circle; assignment counterbalanced across participants) and the unconditioned stimulus (US; electric shock) are represented. The BLA is a locus of sensory convergence and a plausible site for CS-US association in the form of local synaptic plasticity within the amygdala. Intra-amygdaloid circuitry conveys the CS-US association to the central nucleus of the amygdala (CE) that mediates fear responses such as the fear-potentiated acoustic startle. **B.** **Fear conditioning paradigm.** The paradigm consists of a startle habituation, acquisition and extinction phase with a previous quiet baseline period. Acquisition consisted of 5 non-reinforced CS+ presentations and 5 CS+ presentations that co-terminated with the electric shock. The CS− was presented 10 times without reinforcement. Extinction was comprised of 10 non-reinforced presentations of each of the CSs. Before and after acquisition, the subjective US expectancy was assessed. Additionally, contingency awareness was assessed after acquisition.

A differential fear conditioning paradigm with a partial reinforcement schedule was adapted from Schiller et al. [48] (Fig. 1A). The participants were seated in a comfortable armchair (screen distance 1 m) in a dimly lit, sound-attenuated room, connected to a control room via an intercom system. After the attachment of the electrodes, the intensity of the electric stimulation was adjusted for each individual (starting at 1 mA) to a level described as ‘aversive but not painful’. An electrical stimulator (DS-7D, Digitimer Ltd, Hertfordshire, England) was used to deliver the US via Ag/AgCl electrodes to the right lower arm (duration 100 ms). Two equally sized geometrical shapes (yellow square, blue circle) served as the conditioned stimuli (CS+ and CS−, assignment counterbalanced across participants). As the main dependent variable, we measured the acoustic eye blink *startle response.* The startle response is modulated by the amygdala (Fig. 1B) [49], [50] and represents a reliable measure of conditioned fear [51]. The startle stimulus was a 100 dB burst of white noise (50 ms duration, instantaneous rise time) presented binaurally through calibrated headphones. A startle habituation phase included nine startle tones with intertrial intervals (ITI) of 12, 14 or 16 seconds.

#### Acquisition phase

Acquisition comprised pseudorandomized presentations of 10 CS− and 10 CS+ (8 sec. duration), out of which five were immediately followed by the US (50% partial reinforcement) to delay extinction. Startle probes occurred at 4.5 or 7 seconds during 60% of each CS (six CS+ and six CS−) and during 21% of the ITIs (four ITI startles). Prior to acquisition, the participants were instructed to attend to the shapes on the screen and their relationship with the shocks.

#### Extinction phase

The extinction phase started five minutes after the end of the acquisition phase (immediate extinction). The participants were instructed that this phase would be similar to the previous one. The shock electrodes remained in place, but the participants were explicitly instructed that no further electric shock would be applied (instructed extinction). We used instructed extinction to reduce the variance of explicit contingency learning. The trial sequence and startle presentation was identical to the acquisition phase, but no electrical shock was delivered at any time.

#### Expectancy and contingency ratings

Before and after acquisition, US-expectancy was rated on a visual analog scale (‘How much do you expect that this picture will be followed by a shock?’). After the acquisition phase, the CS-US contingency was assessed (‘Please indicate which CS was paired with the shock’) to differentiate between fully aware (both CS+ and CS− identified correctly), partially aware (at least one CS correctly identified), and unaware (no CS correctly identified) participants.

### Physiological recordings and response definition

The electromyogram (EMG) was recorded via two Ag/AgCl minielectrodes (5 mm inner diameter) attached on the Musculus orbicularis oculi below the left eye. The EMG signal was recorded at 1 kHz by a Synamps 1 amplifier (Neuroscan Inc., Charlotte, USA), bandpass filtered between 1–200 Hz, and stored for off-line analysis. The offline analysis in A*vg_Q (* https://github.com/berndf/avg_q [*52*] *)* was comprised of high-pass filtering (65 Hz), rectification, smoothing (40-ms moving average) and removal of slow drifts (2 Hz high-pass filter). Electrode artifacts were removed after visual inspection.

A startle response was classified as valid if the startle response curve first exceeded an individual threshold (3 SDs of a 500-ms pre-startle baseline) and then fell back below the threshold within a latency window of 20–110 ms after startle noise onset. Valid response amplitudes within a fixed target window of 40–90 ms post-stimulus were then averaged across the six CS+ and the six CS− trials with startle tone. Walker and Davis [53] demonstrated that proportional scores are preferable to absolute scores when scoring startle responses. We therefore divided the startle amplitude to each of the CSs by the ITI startle amplitude, thereby expressing CS startle as the proportion of the individual ‘background’ startle level. Since ITI startle amplitudes habituated across the task, combining acquisition and extinction seemed inappropriate (i.e. exaggerating acquisition proportion scores for the CS startles). Phase specific referencing (i.e. of the acquisition CS startles to acquisition ITI startles and extinction CS startles to extinction ITI startles), in turn, would occlude changes from acquisition to extinction. We therefore decided to use the mean ITI startle amplitude during acquisition (showing minimal habituation) as the reference value for CS startle amplitudes during both acquisition and extinction.

### Data analysis

Two separate 2×2, Group (MDD, HC)×CS-type (CS+, CS−), analyses of variance (ANOVA) were computed for the acquisition and extinction phases (main outcome parameter startle response). US expectancy ratings after acquisition were analyzed by paired-sample *t*-tests. Pearson correlations were employed for correlation analyses. The level of significance was set at *p*<.05 (two-tailed). Data analysis was performed using IBM SPSS Statistics 21.

## Results

### Demographic and clinical characteristics

The demographic and clinical characteristics of the participants are presented in Tab. 1. Patients with MDD and healthy controls did not differ in the distribution of sex, age and years in school. MDD patients reported significantly higher levels of depression, stress, sleep complaints and anxiety than healthy controls. Patients with MDD also memorized significantly less digits than healthy controls, pointing to an impairment of hippocampus-dependent, short-term memory. There were no significant differences in attentional performance between the experimental groups (Test for Attentional Performance, TAP).

**Table 1:** Demographic and clinical characteristics.

	MDD Patients (*n* = 37)	Healthy controls (*n* = 40)	*χ^2^/T*	*p*
**Sex (m/f)**	20/17	21/19	*χ^2^* = 0.02	.891
**Age (in years)**	38.4±12.4	37.3±10.5	0.43	.669
**Years in school**	11.8±1.9	12.4±1.2	1.79	.079
**HAMD 21**	28.6±5.7	Not assessed	–	–
**BDI**	29.2±10.5	1.9±2.9	15.34	**<.001**
**PSQ**	67.5±17.0	22.8±15.0	12.26	**<.001**
**ESS**	9.0±3.6	5.1±2.9	5.17	**<.001**
**PSQI**	10.0±4.2	2.6±2.0	9.82	**<.001**
**BAI**	13.8±7.2	1.2±2.0	10.40	**<.001**
**Digit span**	15.1±4.8	17.3±4.1	−2.16	**.034**
**Alertness**	254.4±54.4	246.1±29.5	0.85	.400

The data represent means ± standard deviations. *T*-tests for independent samples and Pearson’s chi-square tests were used. HAMD, Hamilton Rating Scale for Depression; BDI, Beck Depression Inventory; PSQ, Perceived Stress Questionnaire; ESS, Epworth Sleepiness Scale; PSQI, Pittsburgh Sleep Quality Index; BAI, Beck Anxiety Inventory; Digit span, number of correct trials of the Digit Span Test (Hamburg-Wechsler Intelligence Scale for Adults); Alertness, reaction time in ms (TAP). The significant results are given in bold.

### Fear Conditioning

**Figure 2:**
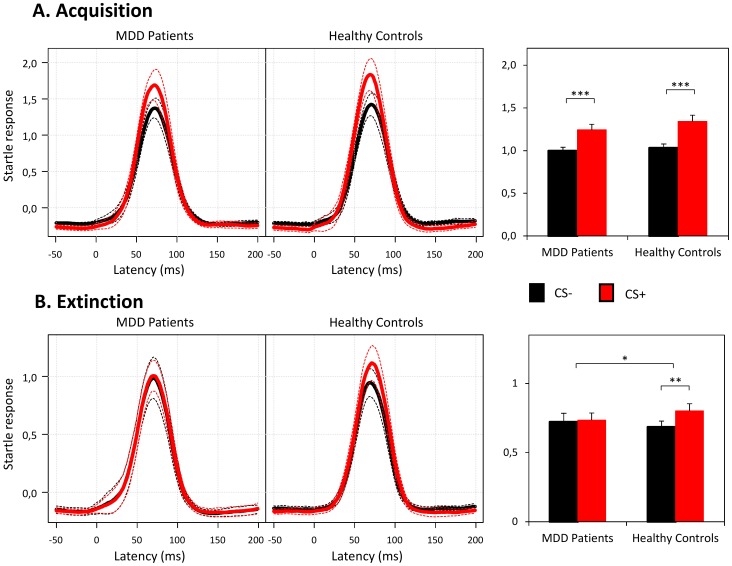
Startle responses to conditioned stimuli. **A.** In the acquisition phase, both groups showed successful fear acquisition, as evidenced by significantly stronger responses to the CS+ than to the CS−. **B.** In the extinction phase, patients with MDD showed significantly stronger extinction learning in comparison to healthy controls, as indicated by the significant interaction between CS type×Group. Particularly, patients with MDD showed robust extinction learning, whereas healthy controls did not. Startle response amplitudes are expressed as a proportion of the mean intertrial interval (ITI) startles during the acquisition phase. Dashed lines represent 95% confidence intervals. Bars represent means ± SEM. Note that the bars represent the mean signal amplitude of the time interval from 40 to 90 ms and not the absolute level, resulting in levels lower than 1. *p<.05, **p<.01, ***p<.001, ANOVA and post-hoc paired-sample *t*-test.

The mean relative amplitudes (CS amplitude divided by ITI amplitude) for both experimental groups for the CS+ and CS− during the acquisition and extinction phase are presented in Fig. 2.

#### Acquisition phase

There was a significant main effect for the factor CS-type (*F*(1, 75)  = 70.72, *p*<.001, *η_p_^2^* = .485), which was indicative of differential responses to the CS+ and CS− in the acquisition phase. No significant main effect for the factor Group *(F*(1, 75)  = 0.78, *p  = *.379, *η_p_^2^* = .010) or CS-type×Group interaction was observed (*F*(1, 75)  = 1.02, *p  = *.316, *η_p_^2^* = .013), revealing similar fear acquisition in both groups. Subsequent paired-sample *t*-tests confirmed successful fear acquisition (CS+>CS−) in both experimental groups (healthy controls: *t*(39)  = −6.60, *p*<.001; patients: *t*(36)  = −5.30, *p*<.001). The strength of conditioning (difference between responses to CS+ and CS−) was not different between the two groups (*t*(75)  = −1.01, *p*  = .316).

#### Extinction phase

As the main finding of the present study the main effect for the factor CS-type (*F*(1, 75)  = 6.31, *p*  = .014, *η_p_^2^* = .078) was modulated by a significant CS-type×Group interaction (*F*(1, 75)  = 4.08, *p*  = .047, *η_p_^2^* = .052), indicating that the strength of extinction learning differed for the two groups. Following up on this interaction, separate paired-sample *t*-tests (CS+ vs. CS−) were computed for each group. Notably, patients with MDD showed complete extinction learning since startle response did not differ between the two CS-types anymore (*t*(36)  = −0.37, *p*  = .717). Healthy controls, by contrast, demonstrated continued and robust differential responding to the two CS-types throughout the extinction phase (*t*(39)  = −3.09, *p*  = .004).

To incorporate acquisition strength in the analysis of extinction learning, we repeated the analysis for the extinction phase including only participants with successful acquisition (response amplitude for CS+>CS−; 31 patients and 35 healthy subjects). Here, the results were even more pronounced. The ANOVA showed again a significant main effect for the factor CS-type (*F*(1, 64)  = 6.31, *p*  = .015, *η_p_^2^* = .090). This effect was modulated by the previously observed significant CS-type×Group interaction *(F*(1, 64)  = 6.23, *p*  = .015, *η_p_^2^* = .089). Two of the patients misidentified both CS+ and CS−. When we repeated the analysis only for participants who were fully aware of the CS-US contingency, the critical CS-type×Group interaction remained significant (*F*(1, 73)  = 4.17, *p*  = .045, *η_p_^2^* = .054).

### US expectancy ratings

Paired-sample *t*-tests showed that neither group differentiated between the CS+ and the CS− in their US expectancy ratings prior to the acquisition phase (*p*>.1). After the acquisition phase, both groups indicated a significantly higher US expectancy towards the CS+ than towards the CS− (healthy controls: *t*(39)  = −14.01, *p*<.001; patients: *t*(36)  = −6.15, *p*<.001).

### Correlation Analyses

Within the group of MDD patients, exploratory Pearson correlations were conducted between the strength of conditioning in the acquisition and extinction phase and clinical characteristics (total duration of illness, average number of episodes, duration of the current episode, average age of MDD onset, BDI score, HAMD score, and BAI score).

**Figure 3:**
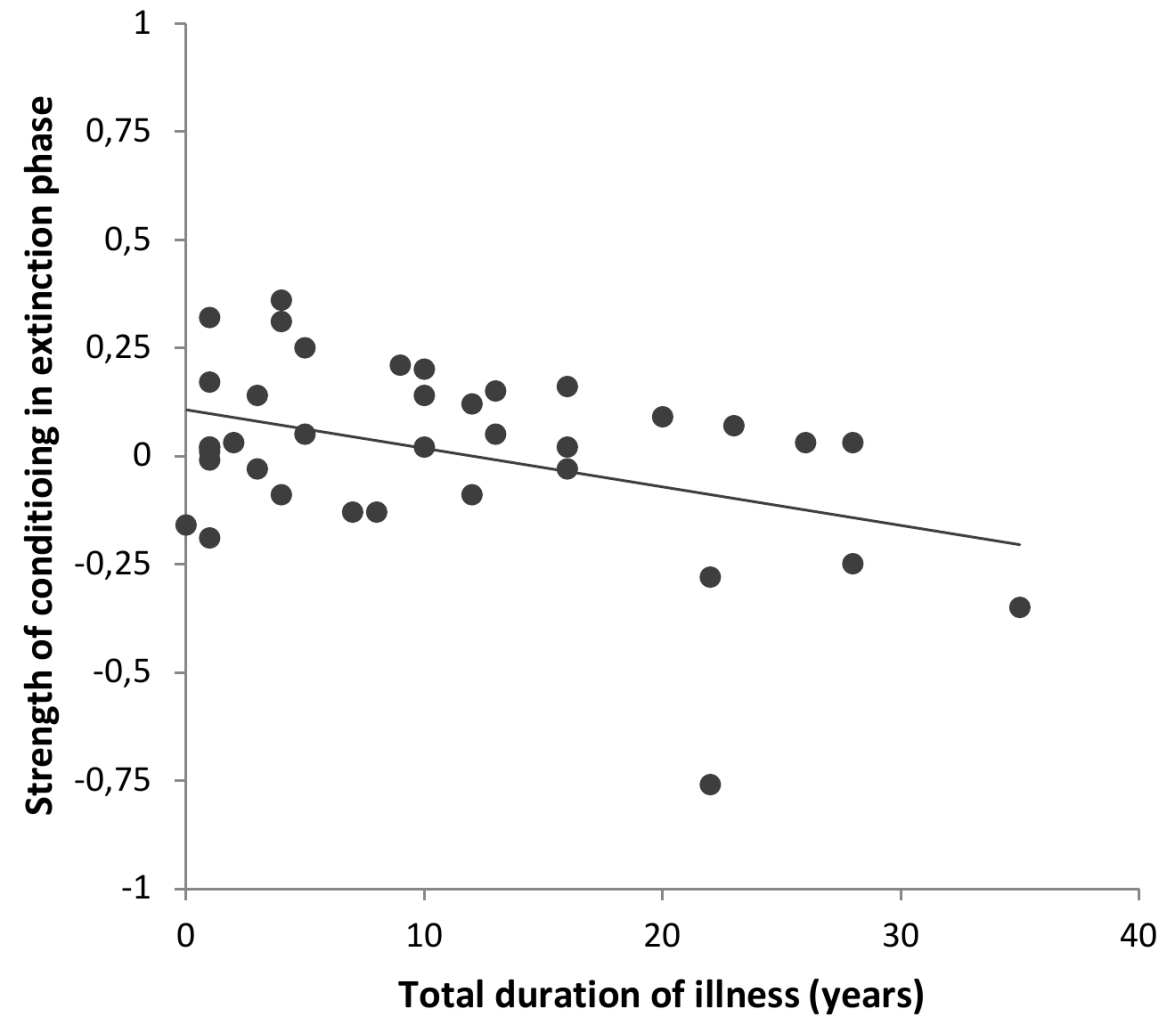
Correlation between the strength of conditioning in extinction and the duration of illness. In the group of MDD patients, the strength of the remaining conditioning in the extinction phase was negatively correlated with the duration of illness, i.e., extinction learning was enhanced in patients with a longer duration of illness, *r*(35)  = −.40, *p*  = .014, Pearson correlation.

In the acquisition phase, a significant positive correlation between the strength of acquisition (difference between CS+ and CS−) and the BDI score was found (*r*(35)  = .41, *p*  = .012), indicating that patients with higher depression scores showed a stronger fear acquisition. There was also a trend for a significant positive correlation between the strength of fear acquisition and the total duration of illness (*r*(35)  = .320, *p*  = .053); i.e., patients with a longer duration of illness tended to show an elevated strength of fear acquisition. In the extinction phase, the strength of remaining conditioning was negatively correlated with the total duration of illness (*r*(35)  = −.40, *p*  = .014), indicating that extinction learning was enhanced in patients with a longer duration of illness (Fig. 3). No other significant correlations were observed (all *p*>.1).

Because the total duration of illness was correlated with the age of the patients (*r*(35)  = .418, *p*  = .010), we added a correlational analysis of age and the strength of conditioning in both phases to rule out the possibility that the observed correlations between the duration of illness and strength of conditioning were primarily driven by age. Age and the strength of conditioning in the acquisition phase were not significantly correlated in patients (*r*(35)  = .145, *p*  = .393). In the extinction phase, a trend was observed (*r*(35)  = −.306, *p*  = .066), revealing that older patients showed enhanced extinction learning. However, in healthy controls, there was no significant correlation between age and the strength of conditioning in the extinction phase (*r*(38)  = .104, *p*  = .524), indicating that extinction learning is not generally enhanced in older participants.

## Discussion

The results of this study are consistent with the hypothesis of enhanced fear extinction learning in patients with MDD compared to healthy controls after comparable levels of acquisition. Based on preclinical work implicating synaptic LTP in the basolateral amygdala as the critical mechanism and site of fear extinction learning [35], [54], our results provide indirect evidence for enhanced amygdala-dependent synaptic plasticity in patients with MDD.

### Fear Extinction

To our knowledge, this is the first study investigating fear extinction learning in MDD. Compared to healthy controls, depressed patients showed enhanced fear extinction learning based on the fear-potentiated eye blink startle response (see Fig. 2). Notably, the neural pathway of the fear-potentiated startle response has been well characterized (for a detailed review please refer to [33]). Only a few synapses involving the cochlear root neurons, neurons in the nucleus reticularis pontis caudalis, and motoneurons in the spinal cord constitute the primary acoustic startle pathway. This pathway is critically modulated by the output of the basolateral amygdala and mediated by the central nucleus of the amygdala (Fig. 1B). Thus, the startle response reflects a fast subcortical process (20–110 ms after the startle-noise onset in a time window prior to any cortex-based motor outputs) and is relatively independent from higher cognitive processes [55].

We propose that an enhancement of synaptic plasticity in the amygdala of depressed patients enables a faster formation of an extinction memory. More specifically, it is thought that acquisition and extinction learning lead to the formation of fear and an extinction memory, respectively [56]–[58]. These memory traces are acquired and stored within distinct but interacting neural networks and compete with one another [58]. Herry et al. [57] identified two distinct populations of basal amygdala neurons: so-called ‘fear neurons’ and ‘extinction neurons’. During the acquisition phase, the fear neurons exhibit an increase in CS+-evoked spike firing that is converted into a CS+-evoked inhibition during the extinction phase. At the same time, extinction neurons show a selective increase in CS+-evoked activity. A possible mechanism in MDD might, therefore, entail faster switching in this activity caused by enhanced amygdala-dependent plasticity. Consistent with this idea, the strength of remaining conditioning in the extinction phase was negatively correlated with the total duration of illness, indicating that extinction learning was enhanced in patients with a longer illness duration (Fig. 3). Future studies using more extinction trials would be informative to further determine potential differences in the speed of extinction learning between the groups.

In contrast to patients with MDD, healthy controls did not show robust extinction learning in the current immediate extinction paradigm. Studies in rats and healthy humans demonstrate that short intervals between acquisition and extinction result in minimal fear suppression – the so-called ‘immediate extinction deficit’ [59]. In the present study, extinction was examined immediately after the acquisition phase – a paradigm that does not induce robust extinction under typical synaptic plasticity conditions, i.e., in controls (e.g., [60], [61]; but see [62]). Altogether, this design and the physiology of the recorded signal indicate basic neural refinements, presumably in the form of synaptic LTP in the basolateral amygdala, as the mechanism driving enhanced fear extinction learning in MDD.

### Fear Acquisition

Both patients with MDD and healthy controls showed robust and similar fear acquisition in a simple fear acquisition paradigm (CS+ vs. CS−) (Fig. 2A). US expectancy and contingency ratings indicated high and comparable levels of cognitive representations of the CS-US association in both groups. This finding is important when focusing on fear extinction as in the present study. In contrast, our previous study used a complex fear acquisition paradigm (two CS+ vs. two CS−) to slow the acquisition process [23]. Here, patients with MDD demonstrated enhanced fear acquisition compared to healthy controls. Interestingly, in the current study, we found a positive correlation between the strength of conditioning in the acquisition phase and the severity of the current depressive episode (BDI score) in patients with MDD. Furthermore, there was a positive correlation between the strength of conditioning and the total duration of illness in the acquisition phase. These observations, although only exploratory in nature, are consistent with the assumption of enhanced amygdala-dependent synaptic plasticity in more severely and longer affected patients with MDD.

### Strengths and Limitations of the Study

To our knowledge, this is the first study on fear extinction learning in MDD. A strength of the study is the large and well-defined sample of patients and controls. With the startle response, we used a widely accepted physiological signal of fear conditioning. In particular, the neural pathway of this signal has been well characterized, identifying the basolateral amygdala as the critical site for fear extinction learning. Furthermore, the selected conditioning paradigm induced robust fear acquisition in both groups and a selective difference in fear extinction learning.

Some study limitations need to be acknowledged. First, all patients with MDD were on antidepressant medications (restricted to stable medication with mainly SSRIs) following standard guidelines for the treatment of severe MDD. However, chronic SSRI treatment has been shown to decrease amygdala activity in animals and healthy humans [63]. Chronic SSRI treatment leads to the reduction of fear acquisition [64], [65] – a possible explanation for why we did not observe a group difference in the acquisition phase. Recent studies have also investigated the effects of SSRIs on fear extinction in rodents, yielding contradictory results depending upon the species tested and the substance examined [66], [67]. In one study, chronic treatment with citalopram impaired extinction learning in rats and downregulated the NR2B subunit of the NMDA receptor in the lateral and basal nuclei of the amygdala [68], which has been shown to be important for synaptic plasticity and fear extinction learning, as a selective blockade of this subunit impairs the acquisition of extinction [69]. Together, these studies indicate that long-term SSRI administration tends to impair amygdala-dependent extinction [66]. This view suggests that the observed enhancement of extinction learning in MDD is not driven by medication status, but by an inherent characteristic of the disorder, and might be underestimated in our sample. Notably, a recent study by Karpova et al. [70] reported the opposite effect, showing that chronic treatment with fluoxetine in C57Bl/6JRcc.Hsd mice caused a facilitation of extinction learning. However, this finding was not replicated in C57BL/6J or 129SI/SvlmJ mice after 21 days of fluoxetine treatment [71].

As a second limitation, it has to be acknowledged that other brain structures, such as the hippocampus, the adjacent dorsal anterior cingulate cortex [72] and the medial prefrontal cortex, that have rich connections with the amygdala are presumably involved in fear conditioning in humans [73], [74] and might therefore have mediated the obtained effects, with the amygdala representing the common final pathway of higher order structures. Thus, brain imaging studies [75] and findings in patients with amygdala lesions [76], [77] indicate that the amygdala is critical for fear acquisition and extinction learning [78]. Unlike hippocampus-dependent memories that undergo system consolidation in the neocortex [79], [80], associative fear memories persist in the amygdala [81].

Third, as an alternative explanation to differences in extinction learning, generalization of fear responses to the CS− during the extinction phase in patients with MDD might have driven the pattern of results. As noted by Lissek et al. [82], in differential fear conditioning tasks it is difficult to distinguish between generalization and differential conditioning. However, in the present study the significant CS-type×Group interaction could not be attributed to any CS-type alone which renders the explanation of fear generalization less probable.

Furthermore, the present analysis focused on relative changes, i.e. the statistical difference in conditioned responding to the CS+ and the CS− in the acquisition and extinction phase. The analysis did not focus on the absolute decrease in startle responses across time, because absolute changes are difficult to interpret, particularly when the experimental context changes (instructed extinction manipulation between the acquisition and extinction phase). We suppose that the (numerical) absolute decrease in startle responses to the CS− for both MDD patients and healthy controls might reflect ongoing habituation effects during the experiment. Last, it is unclear what effect our instructed extinction manipulation had since we had no group without such instructions. This manipulation partially restricts the comparability with studies without such instructions.

### Potential Clinical Significance

Fear extinction learning parallels many aspects of the extinction-based exposure therapy that is widely used as an effective treatment for a number of disorders. Studies on fear extinction in patients with panic disorder, posttraumatic stress disorder (PTSD) or participants with high trait anxiety have demonstrated that these individuals show either reduced extinction (e.g., [83], [84]) or enhanced responses to both CSs during extinction (e.g., [85]). In contrast to this deficiency in extinction, patients with MDD showed enhanced extinction learning in the present study. This finding may be important for optimized treatment protocols. Notably, depressive symptoms did not attenuate the efficacy of exposure (extinction) therapy in patients with PTSD [86], and standard guidelines also recommend exposure therapy in the presence of depression (contraindication psychotic symptoms and acute suicidality) [87]. This is interesting because motivational and cognitive deficits in MDD (see also attenuated hippocampus-dependent short-term memory in the current study) would be expected to instead worsen the efficacy of exposure therapy. In contrast, MDD might represent a brain state of enhanced plasticity in a ventral emotional system, which includes the amygdala, as the neural basis for effective exposure-related extinction learning.

Altogether, the current study provides the first evidence for enhanced fear extinction learning in patients with MDD, which is consistent with the concept of enhanced synaptic LTP in the basolateral amygdala. Future studies are needed to specify the neural mechanisms, to determine the impact of the medication status and to investigate whether enhanced amygdala-dependent plasticity in MDD is a state or trait marker. Clinically, the results propose that MDD might represent a well-suited brain state for exposure-based psychotherapy in many anxiety-related disorders and that the prevalent procedure to first treat depression prior to exposure therapy should be re-evaluated.
